# Tissue-Specific Expression of the Porcine *DHRS3* Gene and Its Impact on the Proliferation and Differentiation of Myogenic Cells

**DOI:** 10.3390/ani15081101

**Published:** 2025-04-10

**Authors:** Jifeng Li, Yong Ruan, Chuanmei Jiang, Jinkui Sun, Dongwei An, Bo Zhou, Huan Liu, Ziyang Li, Houqiang Xu

**Affiliations:** 1 Key Laboratory of Animal Genetics, Breeding and Reproduction in the Plateau Mountainous Region, Ministry of Education, Guizhou University, Guiyang 550025, China; 2 College of Animal Science, Guizhou University, Guiyang 550025, China

**Keywords:** *DHRS3*, tissue expression, myogenic cells, proliferation, differentiation

## Abstract

The *DHRS3* gene, a member of the short-chain dehydrogenase/reductase (SDR) family, is involved in critical metabolic processes in animals. This study investigated the expression patterns of *DHRS3* across various tissues of developmental stages in pigs and preliminarily evaluated its effects on myoblast proliferation, apoptosis, and differentiation. RT-qPCR (real-time quantitative PCR) was employed to analyze *DHRS3* expression in the heart, liver, spleen, lungs, kidneys, longissimus dorsi, foreleg, and hind leg of pigs at 3 days, 6 months, and 12 months of age. Cell proliferation was analyzed using EdU (5-ethynyl-2′-deoxyuridine) assays, RT-qPCR, and flow cytometry, while the expression changes of proliferation-, apoptosis-, and differentiation-related genes were assessed via RT-qPCR. The results indicated that *DHRS3* was expressed in all eight tissues at all three developmental stages. At 3 days, *DHRS3* expression was the highest in the kidneys; at 6 months, it peaked in the liver; and at 12 months, it was again the highest in the kidneys. Across all stages, the liver and kidneys exhibited the highest *DHRS3* expression levels. Functional studies revealed that *DHRS3* overexpression suppressed myoblast proliferation and differentiation while promoting apoptosis. In contrast, *DHRS3* inhibition enhanced myoblast proliferation and differentiation and reduced apoptosis. These findings underscore the regulatory role of *DHRS3* in myogenesis and provide insights into its metabolic and developmental functions in pigs.

## Simple Summary

Dehydrogenase/reductase (SDR) family member 3 (*DHRS3*) is involved in various metabolic processes in animals. During porcine growth and development, *DHRS3* is broadly expressed across multiple tissues, with particularly high levels in the liver and kidneys, suggesting a crucial role in metabolism. Studies on porcine myoblasts indicate that *DHRS3* suppresses myoblast proliferation and differentiation but promotes apoptosis, highlighting its potential regulatory function in muscle development and overall growth.

## 1. Introduction

Skeletal muscle, one of the most abundant tissues in animals, plays a vital role in energy metabolism, locomotion, and respiration. It is also a major determinant of meat yield and quality in livestock and poultry [1,2]. The development and maturation of skeletal muscle constitute a complex biological process where myogenesis critically influences the rate and extent of postnatal muscle growth [3]. Muscle fiber maturation occurs in four stages: myoblast progenitor cells, myoblasts, myotubes, and muscle fibers. The myogenic regulatory factor (MRF) family plays a central role in this process [4,5]. During embryonic development and postnatal growth, myoblasts undergo continuous proliferation and differentiation into myotubes, which subsequently mature into skeletal muscle fibers, forming the functional and structural foundation of skeletal muscle [6].

The short-chain dehydrogenase/reductase (SDR) family constitutes an ancient and extensive group of enzymes that are highly conserved throughout biological evolution and broadly distributed among diverse organisms [7,8,9]. Members of the SDR family share common structural features and play essential roles in various metabolic processes within cells. These processes include carbohydrate, lipid, and steroid metabolism, as well as the maintenance of redox homeostasis—functions closely linked to animal growth, development, and economically significant traits such as reproductive performance [10,11,12]. By facilitating intracellular energy metabolism and biosynthetic pathways, SDR family enzymes exert substantial influence on the development of muscle and skeletal tissues. Their involvement in these critical biological processes highlights the importance of this enzyme family in both fundamental research and practical applications related to animal physiology and productivity [13,14].

Dehydrogenase/reductase SDR family member 3 (*DHRS3*), also known as Rsdr1 and retSDR1, is a highly conserved member of the short-chain alcohol dehydrogenase/reductase (SDR) superfamily, specifically classified within the SDR16C family. *DHRS3* plays a key role in vitamin A (VA) metabolism and regulates various biological processes, including cell proliferation and differentiation [15,16]. In cellular metabolism and redox regulation, *DHRS3* has been shown to participate in intracellular redox reactions. It catalyzes the oxidation–reduction reactions of retinol and steroids, thereby contributing to lipid metabolism and maintaining redox homeostasis [17,18]. Furthermore, studies indicate that the *DHRS3* gene influences myoblast proliferation and differentiation in mice, implicating its role in tissue development and cellular differentiation [19]. In the context of liver development and hepatocyte function, *DHRS3* is expressed in liver tissues where it potentially regulates lipid metabolism and redox balance in hepatocytes. However, the precise molecular mechanisms involved remain unclear and warrant further investigation [20,21].

Skeletal muscle myogenesis is a complex biological process involving distinct stages, including satellite cell activation, myoblast proliferation, and terminal differentiation. This process is regulated by multiple factors [22]. The *DHRS3* gene, which encodes a functional protein, is known to modulate various biological processes by regulating gene expression. However, its specific role and underlying mechanisms in porcine skeletal muscle myogenesis remain poorly understood. This study investigates the expression levels of *DHRS3* in various porcine tissues and examines its potential effects on the proliferation, differentiation, and apoptosis of porcine skeletal muscle myoblasts at the cellular level. By advancing the theoretical understanding of the regulatory mechanisms involved in porcine muscle growth and development, this research provides a basis for elucidating the role of *DHRS3* in muscle proliferation and differentiation.

## 2. Materials and Methods

### 2.1. Animal Sample Collection

The experimental animals were obtained from the Guizhou University Breeding Pig Farm. A total of nine Xiangsu hybrid pigs—three each at 3 days, 6 months, and 12 months of age—were randomly selected under identical feeding conditions and slaughtered. After slaughter, tissue samples were collected from the heart, liver, spleen, lungs, kidneys, longissimus dorsi, foreleg, and hind leg. The samples were immediately frozen in liquid nitrogen and subsequently stored at −80 °C in the laboratory for RNA extraction. In addition, longissimus dorsi samples from 3-day-old pigs were promptly transported to the laboratory for the isolation and culture of myoblasts for further experimentation. The use of experimental animals in this study was approved by the Laboratory Animal Ethics of Guizhou University (EAE-GZU-2022-E047).

### 2.2. RNA Extraction and cDNA Synthesis from Tissue Samples

RNA extraction was conducted using the TRIzol reagent kit (Thermo Fisher Scientific, Waltham, MA, USA) according to the manufacturer’s protocol. RNA concentration (ng/µL) and OD260/280 ratio were measured with a microvolume UV spectrophotometer (NanoDrop 2000, Thermo Fisher Scientific, USA), and RNA integrity was evaluated using 1% agarose gel electrophoresis. The purified RNA was reverse-transcribed into cDNA using the StarScript III Reverse Transcriptase Kit (Genstar, Beijing, China) following the manufacturer’s instructions. To ensure accuracy and prevent contamination, enzyme-free pipette tips, RNAse-free reagents, and sterilized equipment were utilized throughout the process. All procedures were carried out in a laminar flow hood sterilized with ultraviolet light for 30 min.

### 2.3. Primer Design, PCR Amplification, and Sequencing

Primers for real-time quantitative PCR were designed based on the mRNA reference sequences of the pig *DHRS3* gene, along with genes involved in proliferation, apoptosis, and myogenic differentiation, as provided by the NCBI database. Primer design was performed using Primer Premier 5.0 (Table 1), and the primers were synthesized by Beijing Qingke Biotechnology Co., Ltd. (Beijing, China) and stored at 4 °C. For PCR amplification, the reaction mixture was prepared in a total volume of 30.0 µL, consisting of 15.0 µL of 2× Hieff^®^ PCR Master Mix, 1.5 µL of each forward and reverse primer, 1.5 µL of cDNA, and 10.5 µL of ddH_2_O. The PCR program included an initial denaturation step at 94 °C for 5 min, followed by 35 cycles of denaturation at 94 °C for 30 s, annealing at 55–62 °C for 30 s, and extension at 72 °C for 30 s. A final extension was performed at 72 °C for 10 min. PCR products were analyzed using 1.5% agarose gel electrophoresis. Finally, the amplified products were sent to Beijing Qingke Biotechnology Co., Ltd. for sequencing.

### 2.4. RT-qPCR

RT-qPCR was conducted according to the instructions provided with the Real-Time Fluorescent Quantitative PCR Kit (StarScript III, Genstar, China), using GAPDH as an internal reference gene to assess the relative expression of target genes. The RT-qPCR reaction mixture (20 µL) consisted of 10 µL of 2× RealStar Fast SYBR RT-qPCR Mix, 3.2 µL of sterile water, 0.2 µL of each forward and reverse primer, and 0.4 µL of cDNA. The PCR cycling program included an initial denaturation at 95 °C for 2 min, followed by 40 cycles consisting of denaturation at 95 °C for 15 s, annealing at 60 °C for 30 s, and extension at 72 °C for 30 s.

### 2.5. Isolation, Culture, and Induced Differentiation of Porcine Myoblasts

The longissimus dorsi muscle from 3-day-old pigs was dissected, washed twice with 75% ethanol and PBS containing 10% penicillin/streptomycin, and then minced using scissors. The tissue was subsequently placed in DMEM/F12 (Gibco, San Diego, CA, USA) medium supplemented with 1 mg/mL of type II collagenase for digestion. The digestion was carried out in a shaking incubator at 37 °C for 1 to 1.5 h. After digestion, the reaction was terminated by adding an equal volume of a complete medium. The resulting cell suspension was filtered through 100 µm and 70 µm cell strainers and centrifuged at 1500 rpm for 10 min to collect the cells. The cells were washed twice with PBS containing 1% penicillin/streptomycin and resuspended in a complete medium. The suspension was transferred to culture flasks and incubated at 37 °C in a 5% CO_2_ incubator. Two hours later, the cells were purified by differential adhesion [23,24]. During myogenic differentiation, once porcine myoblasts reached confluence, they were induced to differentiate in a differentiation medium containing DMEM/F12 and 2% horse serum for 5 days.

### 2.6. Overexpression Plasmid, shRNA, and Transfection

The coding sequence (CDS) of the pig *DHRS3* gene (GenBank: NM_001105617.2) was amplified based on its sequence available in the NCBI database and subsequently inserted into the pEGFP-C1 vector to construct the overexpression plasmid. Multiple pairs of shRNA targeting *DHRS3* were designed according to the established design principles and synthesized by GEMA Ltd. (Shanghai, China). The interference efficiency was evaluated by RT-qPCR, with the specific shRNA target sequences provided in Table 2. When the cell confluence in a 6-well plate reached 60–80%, 2.5 μg of plasmid DNA or shRNA was transfected into each well. All transfection procedures were carried out following the manufacturer’s instructions for the Lipofectamine™ 3000 reagent (Thermo Fisher Scientific, Waltham, MA, USA). The medium was replaced 6 h post-transfection.

### 2.7. EdU Assay

Cells were seeded into a 96-well plate at a density of 6 × 10^3^ cells per well. After 24 h, plasmids were transfected, and 48 h post-transfection, cells were labeled with EdU working solution and incubated for an additional 2 h. Following the protocol provided with the EdU assay kit (Beyotime, Shanghai, China), cells were then observed and imaged using a fluorescence microscope (Thermo Fisher Scientific).

### 2.8. Flow Cytometry

Following 48 h of transfection, cells were digested with trypsin (Gibco, San Diego, CA, USA) and collected. The cells were then washed twice with PBS by pipetting, and the supernatant was discarded. Next, 1.5 mL of pre-cooled 70% ethanol was added to resuspend the cells, which were then incubated overnight at 4 °C. Fixed cells were centrifuged at 1200 rpm for 5 min, and the supernatant was removed. The cell pellet was resuspended in 2 mL of PBS, followed by centrifugation at 1200 rpm for 5 min and supernatant removal. Cells were then resuspended in 100 μL of RNase A (Beyotime, Shanghai, China) solution. The suspension was incubated in a 37 °C water bath for 30 min. Afterward, 500 μL of propidium iodide (PI) (Beyotime, Shanghai, China) staining solution was added, and the samples were incubated in the dark at 4 °C for 30 min. Finally, cell cycle distribution was analyzed using a flow cytometer (Thermo Fisher Scientific).

### 2.9. Statistical Analysis

Statistical analysis was conducted using SPSS software (version 25.0). In this study, Levene’s test was conducted to assess the homogeneity of variance, and the Shapiro–Wilk test was also employed to evaluate the normal distribution of the data. Differences between the two groups were assessed using the independent samples *t*-test, while one-way analysis of variance (ANOVA) was employed for comparisons involving three or more groups. Data are expressed as means ± standard deviation, with a *p* < 0.05 considered statistically significant. Graphical representations were generated using GraphPad Prism (version 8.0.1).

## 3. Results

### 3.1. Tissue-Specific Expression Analysis of DHRS3 in Pigs

**Figure 1:**
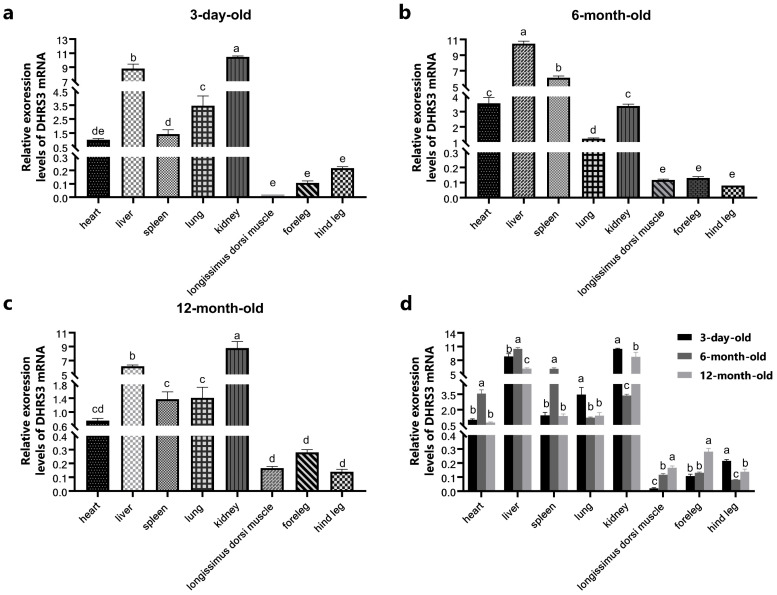
Expression of the *DHRS3* gene in tissues of pigs at different ages. (**a**) Expression of *DHRS3* in different tissues at 0 months of age (3 days); (**b**) expression of *DHRS3* in different tissues at 6 months of age; (**c**) expression of *DHRS3* in different tissues at 12 months of age; (**d**) expression of *DHRS3* in different tissues at different months of age. The data are expressed as mean ± SEM, *n* = 3, Different letters indicate significant differences, and the same letter indicates no significant difference (Tukey’s test).

The relative expression levels of the *DHRS3* gene in tissues from pigs at 3 days, 6 months, and 12 months of age were assessed by RT-qPCR. At 3 days of age, *DHRS3* expression was the highest in the kidneys, followed by the liver and lungs, with the lowest expression observed in the longissimus dorsi muscle (Figure 1a). At 6 months, the highest expression was found in the liver, followed by the spleen, while the lowest expression was observed in the hind legs (Figure 1b). At 12 months, *DHRS3* expression was again the highest in the kidneys, followed by the liver, with the lowest expression in the hind legs (Figure 1c). Across all age groups, *DHRS3* expression was consistently higher in the liver and kidneys, whereas expression in the longissimus dorsi and forelegs increased with age (Figure 1d).

### 3.2. Transfection Efficiency Assessment

**Figure 2:**
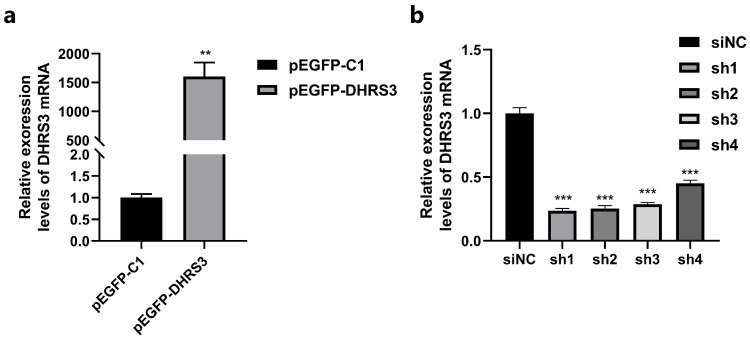
*DHRS3* overexpression and interference efficiency assay. (**a**) The expression level of *DHRS3* after transfection with pEGFP-C1 and pEGFP- *DHRS3* for 48 h; (**b**) the expression level of *DHRS3* after transfection with shNC, sh1, sh2, sh3, and sh4 for 48 h. The data are expressed as mean ± SEM, *n* = 3, ** *p* < 0.01, *** *p* < 0.001 (Dunnett’s test).

The overexpression efficiency of the *DHRS3* gene was evaluated using RT-qPCR, and the most effective shRNA for gene silencing was identified. The results demonstrated that, compared to the control group (pEGFP-C1), the pEGFP-*DHRS3* plasmid significantly enhanced *DHRS3* gene expression (*p* < 0.01) (Figure 2a). Among the four interference sequences tested, all markedly reduced *DHRS3* expression, with sh1 exhibiting the strongest inhibitory effect, making it the candidate for further experiments (*p* < 0.001) (Figure 2b).

### 3.3. DHRS3 Gene Inhibits Proliferation of Porcine Myoblasts

**Figure 3:**
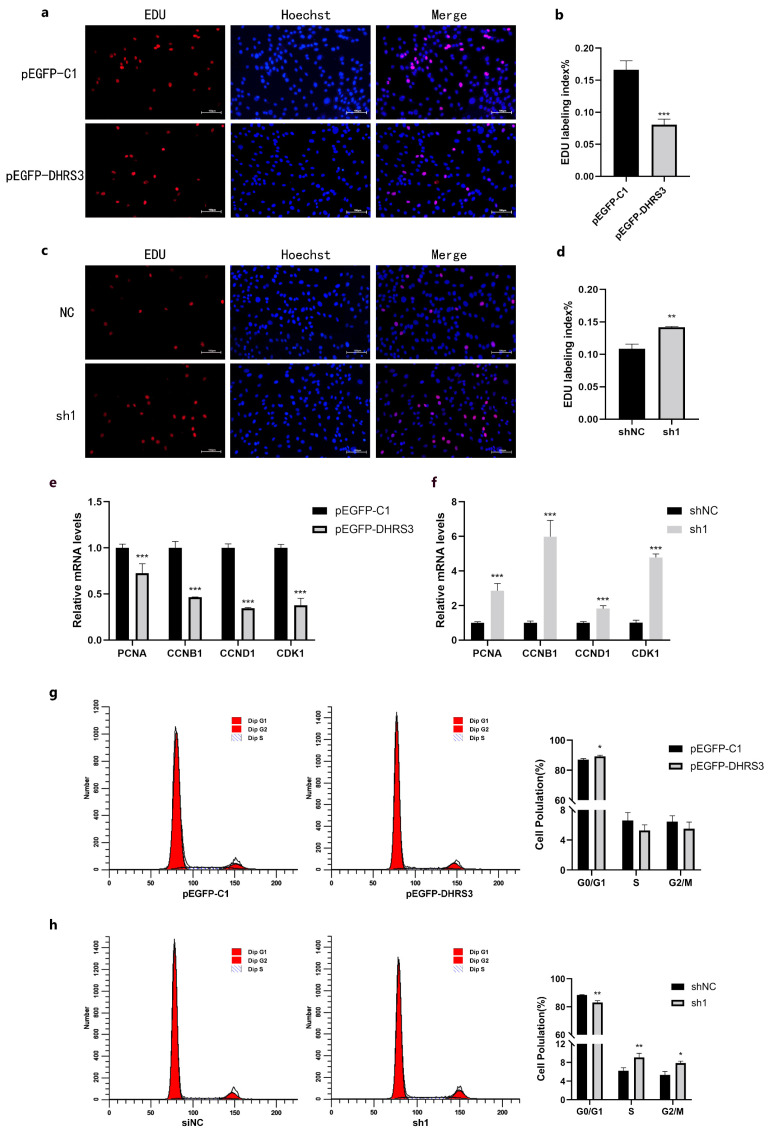
*DHRS3* inhibits pig myoblasts’ proliferation. (**a**–**d**) The EdU results of cell proliferation after transfection with pEGFP-*DHRS3* and sh1 for 48 h; (**e**,**f**) the relative expression of *PCNA*, *CCNB1*, *CCND1*, and *CDK1* after transfection of pEGFP- *DHRS3* and sh1. Flow cytometry was used to examine cell cycle progression following pEGFP-*DHRS3* and sh1. (**g**,**h**) Effects of DHRS3 on myoblast cell cycle. The data are expressed as mean ± SEM, *n* = 3, * *p <* 0.05, ** *p <* 0.01, *** *p <* 0.001 (scale bar, 100 μm).

To ascertain the influence of the *DHRS3* gene on the development of porcine myoblasts, porcine myoblasts were separately transfected with overexpression and interference plasmids of the *DHRS3* gene. Forty-eight hours subsequent to transfection, the impact of the *DHRS3* gene on myoblast proliferation was evaluated through 5-ethynyl-2′-deoxyuridine (EdU) incorporation assay and quantitative real-time polymerase chain reaction (RT-qPCR). EdU staining analysis indicated that overexpression of the *DHRS3* gene notably decreased the quantity of EdU-positive cells (*p* < 0.001) (Figure 3a,b). In contrast, interference with the *DHRS3* gene significantly augmented the number of EdU-positive cells (*p* < 0.01) (Figure 3c,d). RT-qPCR analysis demonstrated that transfection with the pEGFP-*DHRS3* plasmid significantly suppressed the mRNA levels of proliferating cell nuclear antigen (*PCNA*), cyclin B1 (*CCNB1*), cyclin D1 (*CCND1*), and cyclin-dependent kinase 1 (*CDK1*) (*p* < 0.001) (Figure 3e). Conversely, treatment with the sh1 interfering RNA significantly elevated the mRNA levels of *PCNA*, *CCNB1*, *CCND1*, and *CDK1* (*p* < 0.001) (Figure 3f). Flow cytometry was used to examine the cell cycle characteristics of cells transfected with pEGFP-C1, pEGFP-*DHRS3*, NC (negative control), and sh1 (*DHRS3*-targeting shRNA). Compared with the pEGFP-C1 group, the pEGFP-*DHRS3* group showed a significantly higher proportion of myoblasts arrested in the G1 phase (*p* < 0.05), while no significant difference in S-phase cell proportion was observed relative to the pEGFP-C1 control (Figure 3g). Conversely, *DHRS3* knockdown (sh1) led to the opposite trend: a marked decrease in G1-phase cells and significant increases in G2-and S-phase populations (Figure 3h). These results indicate that *DHRS3* knockdown shortens the G1-S transition and promotes cell proliferation, whereas overexpression exerts the opposite effect.

### 3.4. Effects of the DHRS3 Gene on Apoptosis-Related Genes

**Figure 4:**
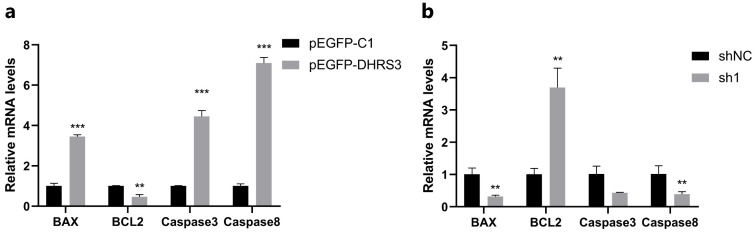
*DHRS3* promotes pig myoblasts’ apoptosis. (**a**,**b**) The relative expression of *BAX*, *BCL2*, *Caspase3*, and *Caspase8* after transfection of pEGFP-*DHRS3* and sh1. The data are expressed as mean ± SEM, *n* = 3, ** *p* < 0.01, *** *p* < 0.001.

To further explore the impact of the *DHRS3* gene on apoptosis in porcine myoblasts, reverse-transcription quantitative polymerase chain reaction (RT-qPCR) was carried out. The results indicated that overexpression of the *DHRS3* gene significantly upregulated the relative mRNA expression levels of apoptosis-associated markers, namely, BCL2-associated X protein (*BAX*), *Caspase 3*, and *Caspase 8* (*p* < 0.001). Concurrently, it downregulated the relative mRNA expression level of the anti-apoptosis gene B-cell lymphoma 2 (*BCL2*) (*p* < 0.001) (Figure 4a). Conversely, when the *DHRS3* gene was interfered with, the relative mRNA expression level of the apoptosis marker *BAX* was significantly downregulated (*p* < 0.05), while that of the anti-apoptosis gene *BCL2* was highly upregulated (*p* < 0.001) (Figure 4b).

### 3.5. The DHRS3 Gene Affects Myoblast Differentiation

**Figure 5:**
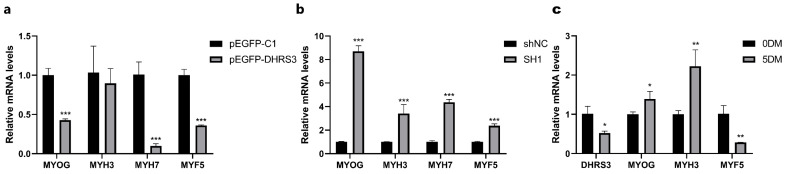
*DHRS3* inhibits pig myoblasts’ differentiation. (**a**,**b**) The relative expression of *MYOG*, *MYH3*, *MYH7*, and *MYF5* after transfection of pEGFP-*DHRS3* and sh1. (**c**) Relative expression levels of *DHRS3*, *MYOG*, *MYH3*, and *MYF5* on Day 0 and Day 5 of induced differentiation. The data are expressed as mean ± SEM, *n* = 3, * *p <* 0.05, ** *p <* 0.01, *** *p <* 0.001.

To explore the role of the *DHRS3* gene in porcine myoblast differentiation, quantitative real-time polymerase chain reaction (RT-qPCR) experiments were conducted. Porcine myoblasts were transfected with *DHRS3* overexpression and suppression plasmids. The results indicated that overexpression of the *DHRS3* gene significantly suppressed the mRNA expression levels of myogenin (*MYOG*), myosin heavy chain 7 (*MYH7*), and myogenic factor 5 (*MYF5*) (*p* < 0.001) (Figure 5a). Conversely, when the *DHRS3* gene was inhibited, the mRNA expression levels of *MYOG*, myosin heavy chain 3 (*MYH3*), *MYH7*, and *MYF5* were highly significantly upregulated (*p* < 0.001) (Figure 5b). Upon replacing the growth medium with differentiation medium to induce myoblast differentiation, we found that *DHRS3* and *MYF5* expression was significantly decreased after 5 days of differentiation (5DM) compared to Day 0 (0DM), whereas *MYOG* and *MYH3* expression was significantly increased (Figure 5c).

## 4. Discussion

Gene expression in various animal tissues reflects the activity of genes within those tissues, providing insights into their physiological functions across different organs [25]. Regulation of gene expression is integral to metabolic pathways as distinct tissues exhibit unique metabolic profiles. Studying gene expression in metabolically active tissues offers valuable information on the tissue-specific regulation of these pathways. Furthermore, analyzing gene expression at different developmental stages helps uncover the molecular mechanisms involved in tissue differentiation and organ formation. Previous research in rats has shown that *DHRS3* is expressed in most tissues, with particularly high levels in the adrenal glands, liver, and ovaries, and moderate levels in the kidneys and lungs. Additionally, other studies have suggested that *DHRS3* in the liver affects vitamin A metabolism and inflammatory responses [26]. In our study, we observed high expression of *DHRS3* in the liver and kidneys of pigs at various ages, indicating that *DHRS3* may play a role in regulating metabolic functions in pigs, which aligns with previous findings.

Muscle formation primarily relies on the proliferation and differentiation of myoblasts. Investigating the proliferation, apoptosis, and differentiation of myoblasts can provide valuable insights into muscle growth and development. To further explore the role of *DHRS3* in porcine muscle development, we assessed the effects of *DHRS3* overexpression and interference on myoblast proliferation, apoptosis, and differentiation.

*PCNA* (Proliferating Cell Nuclear Antigen) is a key marker of cell proliferation, as it promotes cell division by participating in DNA replication and repair [27,28]. Cyclins *CCNB1*, *CCND1*, and the Ser/Thr kinase family member *CDK1* regulate cell proliferation through their roles in the cell cycle [29,30,31]. Specifically, *CCNB1* expression increases during the G2 phase where it forms a complex with *CDK1* to regulate early mitosis [32]. *CCND1* expression is controlled by growth factors and extracellular signals, facilitating the transition from the G1 to the S phase and promoting cell cycle progression [33,34,35]. Previous studies have shown that *DHRS3* inhibits cell proliferation and migration in GC cells, induces G1-phase cell cycle arrest, and promotes apoptosis [36]. In a manganese-induced N27 cell apoptosis model, *DHRS3* expression was downregulated, while miRNA-nov-1 expression was upregulated [37]. Additionally, studies on Circ_*DHRS3* have demonstrated that the knockout of Circ_*DHRS3* enhances cell proliferation and suppresses IL-1β-induced chondrocyte apoptosis [38]. Our results from the EdU assay revealed that *DHRS3* overexpression significantly reduced the number of proliferating cells, while *DHRS3* interference had the opposite effect. Furthermore, overexpression of *DHRS3* led to significantly reduced mRNA levels of proliferation-related genes *PCNA*, *CCNB1*, *CCND1*, and *CDK1*, and a notable increase in the mRNA levels of pro-apoptotic genes *BAX*, *Caspase3*, and *Caspase8*. The mRNA expression of the anti-apoptotic gene *BCL2* was significantly decreased, and these effects were reversed following *DHRS3* interference. These findings are consistent with previous research, suggesting that *DHRS3* inhibits porcine myoblast proliferation and promotes apoptosis.

During myoblast differentiation, the dynamic expression changes of myogenic-specific genes *MYOG*, *MYF5*, *MYH3*, and *MYH7* play crucial regulatory roles in reflecting distinct stages of muscle development. *MYOG* (myogenin), a master regulator of skeletal muscle differentiation, exhibits upregulated expression during early differentiation phase [39]. This elevation activates downstream muscle-specific genes and facilitates the transition from myoblasts to multinucleated myotubes. *MYH3* (myosin heavy chain 3), also known as embryonic myosin heavy chain, predominantly expresses during embryogenesis and participates in early myofiber formation [40]. Its expression typically peaks at initial differentiation stages before being gradually replaced by other myosin isoforms. In contrast, *MYH7* (β-myosin heavy chain) represents the predominant isoform in adult skeletal and cardiac muscles whose expression progressively increases during late differentiation stages, indicating maturation of muscle fibers [41]. *MYF5*, as the earliest expressed myogenic regulatory factor (MRF) in skeletal muscle lineage determination, shows critical involvement in myoblast commitment [42]. In our study, *DHRS3* overexpression significantly upregulated mRNA levels of myogenic markers (*MYOG*, *MYH3*, *MYH7*, and *MYF5*), while gene interference yielded opposite results. This paradoxical observation suggests a potential negative regulatory role of *DHRS3* in myogenic differentiation. Notably, during differentiation induction experiments, both *DHRS3* and *MYF5* expressions exhibited significant downregulation by Day 5 compared to initial stages (*p* < 0.05), whereas *MYOG* and *MYH3* showed marked elevation. This temporal expression pattern implies that *DHRS3* may exert its regulatory function primarily during early differentiation phases, with subsequent downregulation facilitating the progression of myogenic programs. The observed expression dynamics parallel those reported in retinoic acid (RA) signaling pathway-related genes, suggesting *DHRS3* might participate in myogenic regulation through RA metabolic modulation. These findings align with previous studies demonstrating the temporal hierarchy of MRFs in muscle development [43,44].

In this study, we observed that *DHRS3* is expressed in various tissues of pigs, with particularly high expression levels in the liver and kidneys. As the primary metabolic organ and key excretory organs, the liver and kidneys suggest that *DHRS3* may play a crucial role in porcine growth and development. The growth and development of animals rely on the proliferation and differentiation of myoblasts. Through experiments involving *DHRS3* overexpression and interference, we examined its effects on myoblast proliferation and differentiation. Our findings suggest that *DHRS3* may inhibit porcine myoblast proliferation, promote apoptosis, and suppress differentiation. However, the exact mechanisms underlying these effects warrant further investigation.

## 5. Conclusions

In conclusion, this study confirms that the *DHRS3* gene is expressed across various tissues in pigs, with particularly high levels in the liver and kidneys. Cellular experiments revealed that *DHRS3* inhibits porcine myoblast proliferation and differentiation while promoting apoptosis. These findings lay a theoretical foundation for further investigation into the molecular mechanisms regulating myoblast proliferation and differentiation in pigs.

## Figures and Tables

**Table 1:** Gene primers used for RT-qPCR.

Gene	Primer Sequence (5′-3′)	Product Size (bp)	Annealing Temperature (°C)
*DHRS3*	F:AGCACCGAGATGTTTCAGGG	113	60
	R:AGGGTCTGATTGAGCTGCAC		
*GAPDH*	F:TTTGTGATGGGCGTGAACC	171	60
	R:AGTCTTCTGGGTGGCAGTGAT		
*PCNA*	F:AAGTGGAGAACTCGGAAATGGAA	161	60
	R:CTGTAGGAGAGAGTGGAGTGGCTTT		
*CCNB1*	F:GACTGGCTAGTGCAGGTTCAGATG	137	60
	R:ATGGCAGTGACACCAACCAGTTG		
*CCND1*	F:TGACCTGCCTTAGACCTTA	140	60
	R:GCTGCTGTTACTGACTATATC		
*CDK1*	F:GAGCGACGCTGACGTGGTA	72	62
	R:TGGATGTGGTAGATCCCAGCTT		
*BAX*	F:TGACGGCAACTTCAACTGGG	144	55
	R:AGCAGCCGATCTCGAAGGAA		
*BCL2*	F:CAGAGGGGCTACGAGTGGGATG	89	62
	R:CCGGGCTGGGAGGAGAAGATG		
*Caspase3*	F:AGAAGACCATAGCAAAAGGAGCAG	155	60
	R:GTTTGGGTTTGCCAGTTAGAGTTC		
*Caspase8*	F:CTGACCTCTTATTTCACTGGTTCGA	97	62
	R:CTTTCTGGTATTTATCCCCTTGACA		
*MYOG*	F:TGCCCAGTGAATGCAGTTCC	164	60
	R:ATCCTCCACTGTGATGCTGTCC		
*MYH3*	F:ACAGCGGAAAGAAGAAAGTTGC	157	55
	R:CCTGGGGTTTTGGTTTCATT		
*MYH7*	F:GATGCGGAGATGGCCGCATT	171	60
	R:GACTTTGCCACCCTCTCGAGACA		
*MYF5*	F:TGCCAGTTCTCGCCTTCTGAGTA	221	62
	R:GTGGATTTCCTCTTGCACGCTTT		

Note: F stands for upstream primer and R stands for downstream primer.

**Table 2:** The specific shRNA sequences.

shRNA Name	Sequences (5′-3′)
shRNA1	GGACCATGCATGTCCTCATTA
shRNA2	TGATCTATCTGGTGGTGAAAG
shRNA3	CCTTCTCAAGTCCCAGCATAT
shRNA4	ACACCAGCACCGAGATGTTTC
shNC	GTTCTCCGAACGTGTCACGT

## Data Availability

The original contributions presented in this study are included in the article/Supplementary Material. Further inquiries can be directed to the corresponding author(s).

## Supplementary Materials

The following supporting information can be downloaded at: https://www.mdpi.com/article/10.3390/ani15081101/s1, Figure S1: Agarose gel electrophoresis pattern of the CDS region of the porcine DHRS3 gene (a) PCR product of DHRS3 gene in pig; (b) The results of bacterial liquid PCR amplification of the DHRS3 gene. F: TGCCCGACAACCACTACCTGAGC; R: TTGTGAAATTTGTGATGCTATTGC.
