# Supplementary material for: Tissue-Specific Expression of the Porcine DHRS3 Gene and Its Impact on the Proliferation and Differentiation of Myogenic Cells
Source: Animals (Basel). 2025 Apr 10;15(8):1101. doi: 10.3390/ani15081101 (PMC12023973; doi:10.3390/ani15081101)
Supplement: Supplementary file 1 [file animals-15-01101-s001.zip › animals-3582014-supplementary.pdf]

a

b

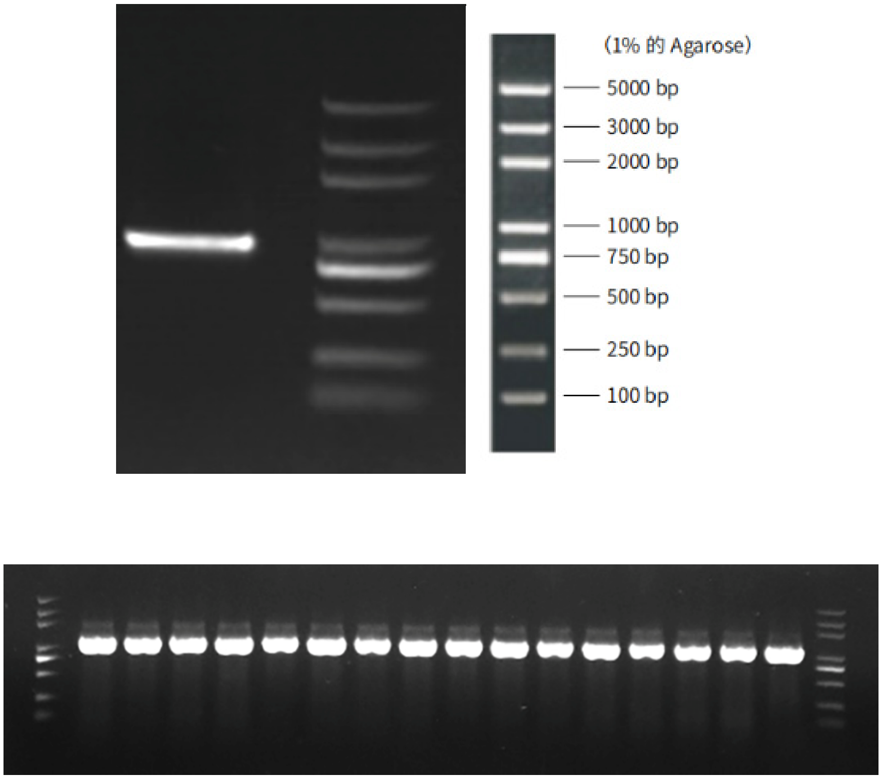

Figure S1. Agarose gel electrophoresis pattern of the CDS region of the porcine DHRS3 gene (a) PCR product of DHRS3 gene in pig; (b) The results of bacterial liquid PCR amplification of the DHRS3 gene.

F: TGCCCGACAACCACTACCTGAGC; R: TTGTGAAATTTGTGATGCTATTGC
